# Innovative Mining of User Requirements Through Combined Topic Modeling and Sentiment Analysis: An Automotive Case Study

**DOI:** 10.3390/s25061731

**Published:** 2025-03-11

**Authors:** Yujia Liu, Dong Zhang, Qian Wan, Zhongzhen Lin

**Affiliations:** 1 School of Art and Design, Guangdong University of Technology, Guangzhou 510090, China; liuyujia@gdut.edu.cn (Y.L.); lzz@gdut.edu.cn (Z.L.); 2 Shenzhen Lingdong Software Development Co., Ltd., Shenzhen 518064, China; zhangdongsite@163.com

**Keywords:** user requirement, data mining, user-generated content (UGC), topic identification, sentiment analysis

## Abstract

As the automotive industry advances rapidly, user needs are in a constant state of evolution. Driven by advancements in big data, artificial intelligence, and natural language processing, mining user requirements from user-generated content (UGC) on social media has become an effective way to understand these dynamic needs. While existing technologies have progressed in topic identification and sentiment analysis, single-method approaches often face limitations. This study proposes a novel method for user requirement mining based on BERTopic and RoBERTa, combining the strengths of topic modeling and sentiment analysis to provide a more comprehensive analysis of user needs. To validate this approach, UGC data from four major Chinese media platforms were collected. BERTopic was applied for topic extraction and RoBERTa for sentiment analysis, facilitating a linked analysis of user emotions and identified topics. The findings categorize user requirements into four main areas—performance, comfort and experience, price sensitivity, and safety—while also reflecting the increasing relevance of advanced features, such as sensors, powertrain performance, and other technologies. This method enhances user requirement identification by integrating sentiment analysis with topic modeling, offering actionable insights for automotive manufacturers in product optimization and marketing strategies and presenting a scalable approach adaptable across various industries.

## 1. Introduction

The automotive industry is undergoing rapid development, with user demands becoming increasingly diverse and personalized [1]. Consumers now focus not only on traditional factors like performance, price, and brand but also on comfort, the fuel economy, technology, and the environmental impact [2]. These complex and evolving demands place greater pressure on automotive manufacturers. These evolving and complex demands present a significant challenge to automotive manufacturers, as they need to understand and respond to them effectively [3]. The timely and accurate identification of user needs is therefore crucial for aligning product offerings with consumer expectations and staying competitive in the market [4,5,6].

User requirement mining systematically collects, analyzes, and interprets user needs, preferences, and expectations [7]. Many studies have proposed various methods for user requirement mining. Traditional methods, such as surveys and focus groups, have long been used to collect data on user needs, but they suffer from limitations such as small sample sizes, high costs, and biases [8]. The rise in social media and user-generated content (UGC) presents a promising alternative, offering large-scale, real-time data that reflect authentic user emotions [9,10,11]. However, the unstructured and noisy nature of social media data creates significant challenges in extracting meaningful insights. Extracting valuable insights from these vast, complex datasets is now a key research issue in user requirement mining [12].

Common topic modeling methods, such as Latent Dirichlet Allocation (LDA) [13], can identify hidden topics in UGC. Yet these methods often struggle with complex semantic relationships and perform inadequately in capturing evolving user needs. Recently, deep learning-based topic modeling has gained traction [14]. BERTopic (Bidirectional Encoder Representations from Transformers for Topic Modeling) [15] combines c-TF-IDF (Class-based Term Frequency-Inverse Document Frequency) and HDBSCAN (Hierarchical Density-Based Spatial Clustering of Applications with Noise) clustering to identify topics in UGC and create rich topic models. This approach is flexible and accurate, effectively handling high-dimensional unstructured data. RoBERTa (Robustly Optimized BERT Pre-training Approach) [16], an advanced pre-trained model for sentiment analysis, captures deeper emotional nuances in user reviews. However, while these techniques are effective in topic identification and sentiment analysis, each approach alone has limitations in capturing user requirements comprehensively [17,18]. The study of comprehensive topic diversity and sentiment depth remains a key challenge in multi-level and multi-context user demand mining. This research seeks to answer the following research question: how can advanced topic modeling and sentiment analysis techniques be combined to more accurately mine and quantify user requirements?

To address this problem, this study proposes a new user requirement mining method that combines BERTopic and RoBERTa. This method identifies primary user concerns in the automotive industry while also quantifying sentiment tendencies. This offers a more refined analysis of user requirements. First, we collect extensive social media data through web scraping. We then apply BERTopic and RoBERTa for topic modeling and sentiment analysis, systematically mining core user needs. By combining these techniques, this study aims to provide a more precise and comprehensive solution for user requirement mining in the automotive industry.

The structure of this paper is as follows: Section 2 reviews user requirement mining and related research on topic modeling and sentiment analysis. Section 3 details our new user requirement mining method. Section 4 presents the empirical results concerning automotive user requirement mining and analyzes the implications. Section 5 further discusses and summarizes the four major types of user requirements, addresses the limitations, and outlines future works. Section 6 concludes the study.

Overall, we find that combining BERTopic and RoBERTa is a beneficial approach to provide a valuable reference for future developments in user demand mining by offering a detailed analysis of both topics and sentiment in UGC, with broad implications for product design and marketing strategies. Our work contributes to the field by (i) proposing a comprehensive approach that integrates topic modeling and sentiment analysis and (ii) demonstrating the application of this novel approach in new areas, offering actionable insights for automotive companies.

## 2. Related Works

### 2.1. User Requirement Mining

In the automotive industry, user requirement mining not only provides a basis for companies to understand consumer behavior and formulate marketing strategies but also offers valuable insights for new product development [19]. Existing approaches to user requirement mining include traditional and emerging methods such as market research, data mining, and social media analysis [20]. Each of these methods, however, has its own limitations. Market research, a conventional method, typically gathers user feedback through surveys and focus group discussions, directly reflecting users’ subjective opinions [21]. However, market research is constrained by small sample sizes, high data collection costs, and results that can be influenced by survey design and implementation, affecting objectivity and accuracy [22]. Data mining mainly relies on structured data, such as sales records and maintenance logs, to analyze user behavior preferences [23]. While this approach is effective in understanding user behaviors, it falls short in capturing subjective experiences and latent needs, particularly regarding sentiment analysis and identifying personalized requirements [24].

In contrast, social media analysis offers more extensive, multi-dimensional user feedback due to its broad data sources and real-time nature [25]. The rise in UGC has provided a new data source for automotive user requirement mining. UGC [26] includes unstructured textual content such as reviews, discussions, and articles posted by consumers on social media, forums, and blogs. This content, generated spontaneously, reflects users’ genuine emotions and experiences, with broad coverage and real-time insights [27]. By analyzing UGC, companies can quickly grasp market trends and identify authentic user evaluations of different models. However, due to the large scale and noisy nature of UGC data [28], efficiently extracting valuable insights remains a major challenge.

The rapid development of natural language processing (NLP) provides effective tools to address this challenge. NLP automates the processing of large-scale unstructured text, extracting key information and sentiment tendencies [29]. Common text analysis methods, such as TF-IDF (term frequency–inverse document frequency) [30,31], measure the importance of words based on their frequency in documents and across corpora, extracting key topics. The introduction of deep learning models, particularly pre-trained models like BERT (Bidirectional Encoder Representations from Transformers), has further improved the precision and effectiveness of sentiment analysis by capturing complex contextual semantics in the text [32]. However, despite significant progress in NLP, efficiently handling noise, dynamic sentiment variations, and diverse requirements in UGC data remains an unresolved research issue [33].

### 2.2. Topic Modeling and Sentiment Analysis

In user requirement mining, topic modeling and sentiment analysis are two key research directions [34]. Each provides different levels of understanding of user feedback, and their combination offers a more comprehensive perspective for multi-dimensional user requirement mining. Topic modeling focuses on identifying latent topics from large-scale text data [35], while sentiment analysis reveals user attitudes by examining emotional expressions within the text [36]. This combined approach not only uncovers core topics of interest to users but also quantifies their sentiment toward these topics, supporting companies in formulating more targeted marketing strategies.

The theoretical foundation of topic modeling originates from statistics and information retrieval [37]. It aims to automatically identify latent topics by analyzing word co-occurrence patterns in text data [38]. Traditional topic modeling methods, such as Latent Dirichlet Allocation (LDA) [13], perform well on relatively static text data but face challenges with dynamic and complex datasets. Recently, deep learning-based methods like BERTopic, which combine contextual embeddings and density clustering techniques, have significantly improved the accuracy and flexibility of topic identification [39,40]. These advancements enable researchers to capture nuanced semantics in the text, identifying users’ true needs.

Complementing topic modeling, sentiment analysis focuses on detecting emotional tendencies within the text, typically classifying sentiments as positive, negative, or neutral [41]. The theoretical basis of sentiment analysis draws from psychology and linguistics, using sentiment lexicons or machine learning models to quantify emotional information in the text [42]. The application of deep learning models like RoBERTa has further enhanced sentiment classification accuracy, allowing for a better grasp of contextual relations and emotional complexity within the text [43]. Nonetheless, the diversity of emotional expressions and the ambiguity of contexts remain ongoing challenges in sentiment analysis.

In summary, while research on topic modeling and sentiment analysis has advanced considerably, studies combining these two methods are still limited. Therefore, this study proposes a novel approach to user requirement mining by integrating BERTopic and RoBERTa, aiming to combine topic modeling with sentiment analysis for a more comprehensive understanding of user needs.

## 3. A User Requirement Mining Analysis Method Based on BERTopic and RoBERTa

**Figure 1:**
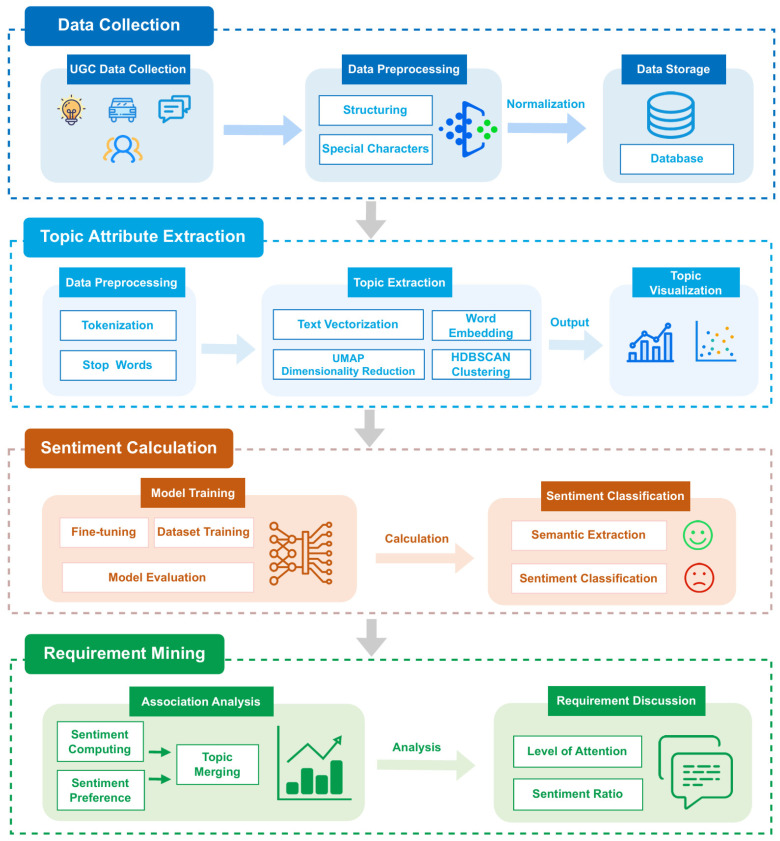
User demand mining framework based on BERTopic and RoBERTa.

This study presents an analysis method that combines the BERTopic and RoBERTa models using UGC data, aimed at deeply exploring and analyzing the multi-dimensional characteristics of user requirements for automobiles. This method enables us to effectively identify and summarize key topic attributes that users care about, thereby precisely refining their specific needs. The technical framework is illustrated in Figure 1, which outlines the complete process from data collection to requirement analysis.

As shown in Figure 1, the main steps include the following:

(1) UGC Collection: We use Python (3.10) to develop a web scraping program that collects UGC related to automobiles from Chinese media platforms such as Autohome, Dongchedi, Weibo, and Zhihu. The data are processed using regular expressions to filter and extract content containing automotive keywords (e.g., “automobile”, “model”, “fuel consumption”, and “power”). After preliminary screening, the data undergo cleaning and preprocessing before being stored in a database for subsequent analysis;
(2) Topic Attribute Extraction: Upon the completion of data preprocessing, we conduct feature extraction to obtain attribute information for each comment. The c-TF-IDF algorithm is employed to assess the importance of words within documents and convert the text into high-dimensional vector representations. Additionally, the RoBERTa model is utilized for the semantic encoding of feature vectors, capturing the deep semantic information of the text. Finally, the BERTopic model performs clustering analysis on the text to identify the main attributes of user comments;
(3) Sentiment Calculation: After attribute extraction, we conduct sentiment analysis to identify the sentiment polarity and intensity of user comments. This involves constructing a sentiment lexicon and training a sentiment classification model to categorize the text into positive and negative sentiments. We combine this with the data encoded by RoBERTa for sentiment polarity determination, calculating the sentiment value for each text to obtain the sentiment distribution for each attribute cluster;
(4) Requirement Mining: Following the completion of sentiment calculation, we conduct a comprehensive analysis of attribute clusters and sentiment values to systematically summarize user focus points and requirements. First, we calculate user attention to different attributes based on quantity and sentiment intensity. Next, we analyze specific evaluations and sentiment tendencies of users toward these attributes. Finally, we identify emerging needs and potential market opportunities, providing a scientific basis for brand growth in the automotive industry.

### 3.1. BERTopic Identification

To better capture the core semantic information within the text, this paper employs the BERTopic model to identify text themes. Additionally, semantic embedding, dimensionality reduction techniques, and advanced clustering algorithms (HDBSCAN) are utilized to ensure the accuracy of theme extraction and the model’s generalization capabilities. The following subsections detail the main technical components of BERTopic and their roles in topic modeling.

#### 3.1.1. c-TF-IDF-Based Keyword Extraction

BERTopic (Bidirectional Encoder Representations from Transformers for Topic Modeling) is an advanced topic modeling tool that combines the BERT pre-trained model with the c-TF-IDF (Class-based Term Frequency-Inverse Document Frequency) method [15]. This combination allows for the precise identification of potential themes within textual data. The c-TF-IDF metric calculates the importance of terms across an entire category or theme, rather than within a single document. Specifically, it computes term frequencies at the category level and combines them with inverse document frequencies within that category, providing a more accurate metric. This capability enables c-TF-IDF to better capture the differences between various themes, enhancing BERTopic’s reflection of the semantic structure in the text when generating topic models.

By applying c-TF-IDF, BERTopic can identify core themes in textual data at a higher abstract level, generating semantically coherent topic models that provide more precise topic extraction for text analysis. This method not only improves the accuracy of topic modeling but also facilitates a deeper and more effective analysis of user-generated content.

#### 3.1.2. Semantic Embedding Representation

Semantic embedding is a method that maps the semantic information of the text into a high-dimensional vector space, capturing deep semantic relationships among words, phrases, or sentences. In BERTopic, semantic embedding primarily relies on the embedding representations of the pre-trained language model BERT. BERT processes text using a bidirectional Transformer architecture [44], enabling it to comprehend a sentence’s context from both left to right and right to left, thereby generating richer semantic information. This characteristic allows it to effectively capture the complex semantic associations between words and sentences, especially in scenarios involving long texts and complex contextual information. By mapping the text into a high-dimensional vector space, BERTopic establishes a precise semantic foundation for subsequent topic modeling.

#### 3.1.3. UMAP Dimensionality Reduction

Text embedding representations typically have very high dimensions, making direct clustering analysis computationally complex and prone to noise. UMAP (Uniform Manifold Approximation and Projection) is a popular nonlinear dimensionality reduction technique [45] designed for high-dimensional data processing. UMAP effectively preserves both the local and global structures of data, ensuring that the reduced dimensionality data still reflects the topological relationships of the original data. Through UMAP’s dimensionality reduction, the dimensions of semantic vectors are significantly reduced while retaining the original features of the text, providing a more effective input for subsequent clustering analysis.

#### 3.1.4. HDBSCAN Clustering Method

HDBSCAN is a density-based clustering algorithm [46] that extends the DBSCAN algorithm to hierarchical clustering, allowing for the extraction of planar clusters based on clustering stability. The core advantage of HDBSCAN lies in its ability to handle clustering problems with varying densities and its greater robustness to parameter selection. Unlike traditional clustering algorithms such as K-means (k-means clustering algorithm), HDBSCAN can automatically determine the number of clusters without prior specification. This feature makes it more flexible in practical applications. Moreover, HDBSCAN effectively handles noise and outliers by identifying and ignoring noisy points during clustering, and it is capable of discovering clusters of arbitrary shapes. These advantages make HDBSCAN an ideal clustering choice in the analysis of textual data.

### 3.2. RoBERTa Sentiment Analysis

This section systematically elucidates the key mechanisms of the RoBERTa model in sentiment analysis tasks. We will begin with the bidirectional Transformer architecture of RoBERTa, exploring how it precisely captures sentiment information in the text through a self-attention mechanism. Additionally, this section will analyze the optimization processes of its pre-training and fine-tuning strategies, the core role of the [CLS] token in semantic extraction, and how RoBERTa implements sentiment classification predictions through the Softmax layer (an activation function used in neural networks).

#### 3.2.1. Bidirectional Transformer Architecture of RoBERTa

RoBERTa’s bidirectional Transformer architecture excels in sentiment analysis tasks, primarily due to its ability to process the text from both left to right and right to left, capturing rich contextual information [47]. This bidirectionality allows RoBERTa to understand the sentiment tendencies of the text more accurately. Its self-attention mechanism assigns different weights to each word, highlighting vocabulary that is crucial for emotional expression. For example, transitional words like “but” can often change the emotional direction of an entire sentence; RoBERTa effectively captures these subtle changes through self-attention.

Moreover, RoBERTa’s multi-layer Transformer structure extracts deep semantic meanings of the text in a layer-by-layer fashion, not only improving the accuracy of sentiment classification but also effectively identifying complex emotional expressions. Compared to BERT, RoBERTa undergoes significant optimizations, such as the removal of the next sentence prediction (NSP) task and adjustments to training parameters, enabling the model to focus more on the internal emotional cues of individual sentences. These optimizations not only enhance training efficiency but also improve the model’s precision and generalization capability in sentiment classification, making it particularly effective for analyzing the sentiments in UGC data.

#### 3.2.2. Pre-Training and Fine-Tuning Mechanism

The pre-training and fine-tuning mechanisms of RoBERTa are crucial for its outstanding performance in sentiment analysis tasks. During the pre-training phase, RoBERTa undergoes self-supervised learning on large-scale unlabeled text datasets by utilizing an improved masked language model (MLM) task [48]. In this task, the model learns contextual relationships by predicting randomly masked words, thus capturing rich language representations. RoBERTa’s pre-training includes multiple optimizations, such as a larger dataset, longer training periods, larger batch sizes, and higher learning rates, significantly enhancing its ability to understand complex semantics.

In sentiment analysis tasks, the pre-trained RoBERTa model requires fine-tuning. During the fine-tuning phase, the model is supervised with a labeled sentiment classification dataset. In this process, RoBERTa’s parameters are adjusted according to the specific task, allowing it to exhibit better classification performance on domain-specific data. By adding a Softmax classification layer, the model can predict the sentiment categories (such as positive, negative, or neutral) based on the feature representations of the text. The fine-tuning mechanism enables RoBERTa to effectively apply the general language knowledge acquired during pre-training to specific tasks like sentiment analysis, significantly improving classification accuracy and generalization capability.

#### 3.2.3. Semantic Extraction Function of the [CLS] Token

In RoBERTa’s architecture, a special [CLS] token (classification token) [49] is introduced at the beginning of each input text sequence. This token is crucial for the semantic summarization of the entire text sequence and plays a key role in classification tasks. In sentiment analysis, RoBERTa uses the [CLS] token to extract the semantic representation of the entire sentence and determine the sentiment tendency of the text based on this representation. Specifically, after processing through multiple Transformer layers, the [CLS] token contains the global contextual information of the entire text sequence. Each word’s features interact with those of other words through the self-attention mechanism, compressing the semantics of the entire text into the output vector of the [CLS] token.

For sentiment classification tasks, the model predicts the sentiment category (such as positive, negative, or neutral) based on the vector representation of the [CLS] token through a fully connected layer and a Softmax classifier. The advantage of this mechanism lies in RoBERTa’s ability to gain a global semantic understanding through the [CLS] token, rather than making sentiment judgments word by word, thereby integrating the various parts of the sentence. This approach not only improves the precision of sentiment classification but also better captures subtle emotional changes within complex sentences.

#### 3.2.4. Softmax Layer for Sentiment Classification

In RoBERTa’s sentiment analysis tasks, the Softmax layer is responsible for transforming the feature representations generated by the model into specific sentiment category predictions. The Softmax layer is situated at the final layer of the model, receiving the feature vector from the [CLS] token and mapping it to a probability distribution over various sentiment categories. Specifically, the Softmax layer applies a linear transformation to the output vector from RoBERTa, converting it into an output dimension equal to the number of sentiment categories. Each dimension corresponds to a sentiment category, such as positive, negative, or neutral. The Softmax function then converts these output values into a probability distribution, representing the relative likelihood of each sentiment category. The core function of this layer is to process the output values through an exponential function and normalize them, ensuring that the probabilities of all categories sum to one.

The role of the Softmax layer is to map the high-dimensional feature representations generated by the model into a probability distribution that is easy to interpret and make decisions about, allowing the model to output specific sentiment category predictions. By minimizing the cross-entropy loss (cross-entropy loss is a loss function commonly used in classification tasks) between the predicted probabilities and true labels during training, the model continuously optimizes its parameters, improving classification accuracy. This method ensures that RoBERTa can accurately determine the sentiment tendencies of complex texts and make reliable predictions.

## 4. Empirical Method

This study aims to systematically uncover automotive user needs by analyzing UGC data to explore users’ emotional tendencies and focal themes in the automotive domain. To achieve this goal, we first employed a customized Python web scraping program to collect data from various major platforms. After processing the collected data, we utilized the BERTopic model for topic extraction to identify the important themes that users are concerned about. Additionally, we integrated a fine-tuned RoBERTa model for sentiment analysis to comprehensively assess users’ emotional tendencies. Ultimately, through the comprehensive application of these techniques, we aim to provide deep insights into the characteristics of automotive user needs and emotional attitudes, offering robust data support for optimizing automotive product design and marketing strategies.

### 4.1. UGC Data Collection

Data collection is a crucial starting point for mining user needs. To ensure the broadness and diversity of the research, this study adopted a multi-source data collection strategy by selected four major platforms that generate UGC data: Autohome, Dongchedi, Weibo, and Zhihu. These platforms have a large user base and high activity levels, covering a wide range of consumer evaluations and discussions in the automotive field, thereby providing rich and multi-dimensional data sources for the research. By aggregating data from multiple sources, this approach broadens UGC coverage and mitigates biases from single-source reliance, thereby improving the robustness and representativeness of the findings.

A customized web scraping program was developed in Python, with different crawling strategies and parameters designed for each platform to ensure the comprehensiveness and representativeness of the data collected. Specifically, the web scraper defined key fields to be extracted, covering the main content of user evaluations, including review texts, publication dates, user IDs, the number of likes, and the number of comments. To facilitate subsequent integration, each dataset was formatted to maintain consistent field definitions across sources. Table 1 presents the core fields required for data extraction in this study.

In the Python-based web scraper design, third-party libraries such as Requests, Selenium, Beautiful Soup, and Lxml were employed for data retrieval and parsing. Additionally, scheduled tasks were configured to regulate the request frequency, ensuring the automated and efficient daily collection of automotive-related content across platforms. To ensure the accuracy of relevance of the collected data, regular expressions (regexs) [50] were utilized during the preprocessing stage to filter the initially acquired UGC data. This approach efficiently identify and extract content related to automobiles while removing irrelevant information, thus ensuring the purity and consistency of the topics in the dataset. The design of regex keywords took into account the diversity of user discussions, covering common automotive brands, models, components, and other related terms. A sample of the regex keywords used is shown in Table 2.

After filtering automotive-related keywords using regular expressions, further rigorous data cleaning was performed to retain only relevant UGC and usable texts. The cleaning process included the removal of HTML tags, special characters, emojis, punctuation, and stop words to refine the textual data. Additionally, each dataset underwent deduplication, anomaly detection, and consistency checks to ensure data accuracy and completeness.

Ultimately, a total of 56,467 valid data entries were collected and cleaned. This data collection and cleaning process ensured that the user-generated content obtained had broad representativeness, covering various aspects of automotive product evaluations. The data laid a solid foundation for subsequent topic modeling and sentiment analysis, ensuring the reliability and practicality of the research results. Table 3 displays samples of the cleaned data.

### 4.2. Topic Identification Using BERTopic

This section employs the BERTopic model to extract themes from UGC data in order to identify significant topics of interest among users. Initially, the text data undergo preprocessing, which includes tokenization and stopword filtering. The tokenization step aims to segment the text into individual words, while stopword filtering focuses on removing common words with little substantive meaning (e.g., “的”, “了”, and “在”) to minimize the impact of noise on theme extraction.

Subsequently, we utilized the BERTopic library in Python to train the model and extract themes. First, pre-trained BERT models convert the text data into embedding representations that capture deep semantic information. Next, we apply UMAP for dimensionality reduction, followed by HDBSCAN for clustering analysis. Through repeated experimentation, we set the minimum cluster size (min_topic_size) to 20 and allowed the number of topics (nr_topics) to be determined automatically. The application of the BERTopic model to the automotive UGC dataset ultimately identified 297 themes.

**Figure 2:**
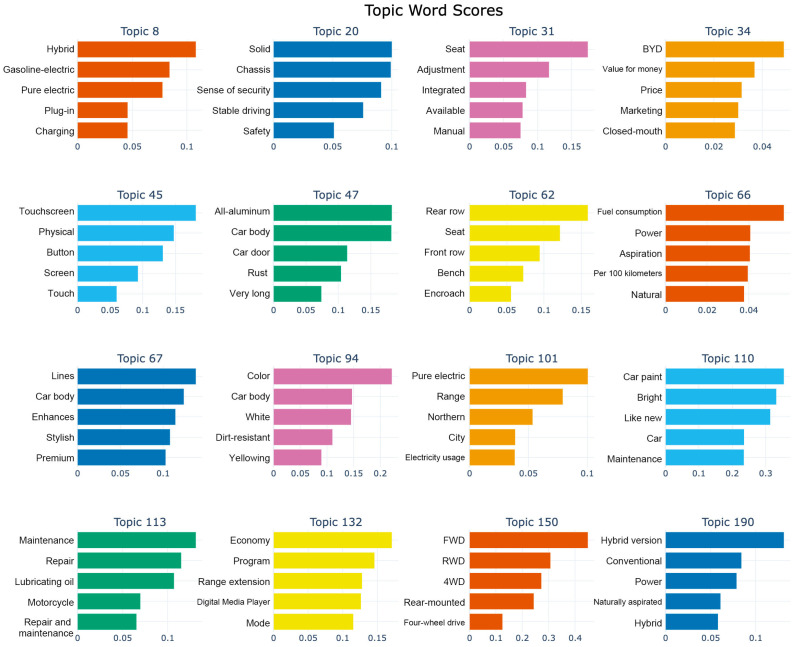
Distribution of topic-specific keywords reflecting user interest in the automotive industry.

Figure 2 illustrates some of the themes identified by the model along with their keywords, visually reflecting the frequency distribution of keywords within each theme, thereby facilitating the rapid identification of important terms in specific topics. Each set of bars in the same color represents a group of related themes and their associated keywords. The horizontal axis indicates the score of each term within the specific theme; a higher score suggests a stronger relevance of the term to that theme. The vertical axis lists the various keywords across different themes. These identified themes reflect the main points of interest for users when discussing automobiles, covering various aspects. For example, Topic 8 focuses on vocabulary related to the powertrain, such as “hybrid”, “petrol-electric”, and “pure electric”, indicating users’ interest in different power types. Topic 20 addresses automotive safety and comfort, with keywords like “seats”, “adjustment”, and “safety”, highlighting users’ emphasis on the riding experience and safety assurance. Other themes follow this pattern.

**Figure 3:**
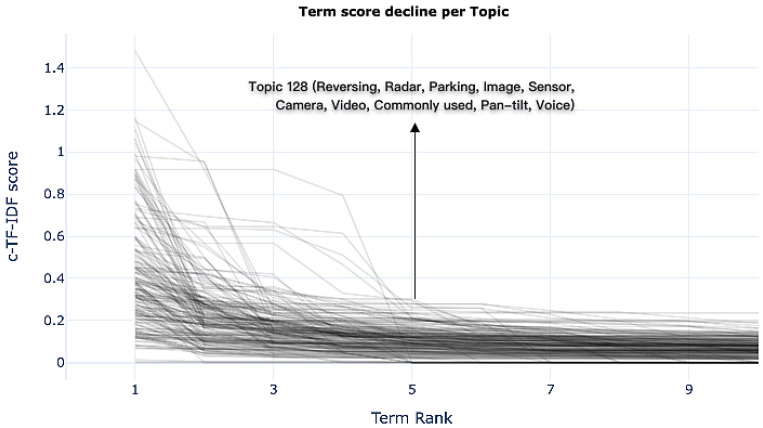
Trend of term score decline by topic.

Figure 3 shows the terms with the highest c-TF-IDF scores in each theme and their importance, revealing the core keywords of the themes. The horizontal axis ranks the terms within a specific theme, while the vertical axis represents the probability scores of those terms within the theme. The curve illustrates how the importance of the terms declines as their rank increases. For instance, in Topic 128, the top keywords include “reversing”, “radar”, “parking”, “image”, “sensor”, “camera”, “video”, “common”, “gimbal”, and “voice”, collectively reflecting user interest in reversing assistance and related intelligent technologies. The c-TF-IDF scores of the top five to seven keywords are relatively higher than those of other words, demonstrating their key significance within the theme. Therefore, these high-frequency terms not only aid in quickly identifying the core content of the themes but also further reinforce the user’s focus on specific needs. Based on this analysis, subsequent theme interpretations will prioritize the top seven high-frequency keywords to gain deeper insights into users’ actual needs.

**Figure 4:**
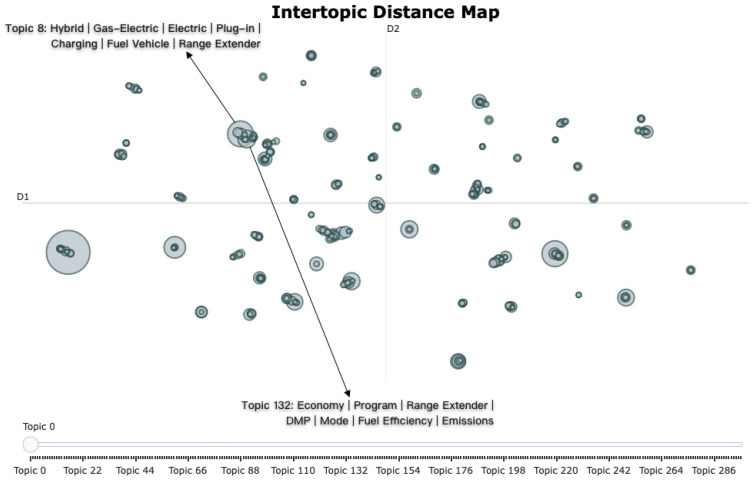
Intertopic distance map. Each circle in the map represents a theme, with the distance between circles reflecting the semantic similarity of the themes. The size of the circles may indicate the relative significance or category of the themes. Circles positioned closely together signify a high degree of content relevance, highlighting overlaps in keywords and discussion topics.

Figure 4 visualizes the similarities and relationships between themes, displaying the positions of various themes and their semantic structures in a two-dimensional space. Each circle represents a theme, with the distance between circles reflecting the semantic similarity of the themes, while the size of the circles may suggest different categories of themes. Circles that are close together indicate a high degree of relevance in content, reflecting overlaps in keywords and discussion topics. For example, Topic 8 includes keywords such as “hybrid”, “petrol-electric”, “pure electric”, “plug-in”, and “charging”, which primarily revolve around the driving methods and power types of new energy vehicles, indicating users’ concerns about multiple power choices. Its neighboring topic, Topic 132, encompasses keywords like “economy”, “extended range”, “fuel-efficient”, and “emissions”, focusing on the economic, environmental, and energy efficiency aspects of new energy vehicles. The content discussed in both themes is highly correlated, aligning with user interests in the performance of new energy vehicle technologies and their practical usage costs.

### 4.3. Sentiment Analysis with RoBERTa 

In this section, we utilized the pre-trained RoBERTa model as a sentiment classifier via the Hugging Face Transformers library (a popular open-source library that provides state-of-the-art machine learning models for NLP), with BERT also included as a benchmark for comparison to comprehensively evaluate RoBERTa’s performance in sentiment analysis. We fine-tuned both models on a sentiment analysis dataset sourced from Weibo, which contains 100,000 labeled instances, comprising 50,000 positive and 50,000 negative samples. To ensure effective model training, we split the dataset into training and validation sets in a 9:1 ratio. Detailed training parameters are provided in Table 4.

The training parameters were selected based on empirical best practices and computational efficiency. A batch size of 128 was adopted to maintain stable gradient updates without exceeding memory constraints. The learning rate was set to ${5\times 10}^{-5}$, a widely used configuration for fine-tuning transformer-based models, ensuring a balance between convergence speed and stability. The number of training epochs was limited to four to mitigate the risk of overfitting, and the hidden size of 768 aligns with the standard architecture of both models, preserving consistency in feature representation.

Upon completing model training, we assessed the performance of both RoBERTa and BERT on the validation set using four key metrics: accuracy, precision, recall, and the f1-score. The specific formulas for these metrics are as follows:

(1) $$Accuracy=\frac{TP+TN}{TP+TN+FP+FN}$$

(2) $$Precision=\frac{TP}{TP+FP}$$

(3) $$Recall=\frac{TP}{TP+FN}$$

(4) $$F1=2\times \frac{Precision\times Recall}{Precision+Recall}$$

Here, TP represents the true positive count, TN the true negative count, FP the false positive count, and FN the false negative count. The F1-score balances precision and recall, serving as an indicator of classification quality, especially when classes are imbalanced.

Following the training and evaluation of the RoBERTa and BERT models, we obtained the performance comparison results for sentiment analysis on the Weibo dataset; the results are summarized in Table 5. The comparison shows that RoBERTa outperformed BERT across all evaluation metrics, particularly in accuracy and recall, indicating RoBERTa’s superior ability to capture sentiment information and better understanding of sentiment tendencies in user-generated content.

Upon completing model training and validation, we input the preprocessed UGC text data into the trained RoBERTa model for sentiment classification, assigning each text sample a sentiment label as either “positive” or “negative.” These sentiment labels are then integrated with the thematic information extracted from the BERTopic model for a sentiment distribution analysis within each topic. By linking sentiment classification with theme modeling results, we gain deeper insights into users’ sentiment tendencies across different themes, revealing their attitudes and feedback towards automotive brands, features, or services. This provides a foundation for better understanding user needs and optimizing product design.

### 4.4. Correlation Analysis Between Sentiment and Topics

Following the completion of topic modeling and sentiment analysis, this study obtained two key datasets: the “Sentiment Distribution” and the “Topic Distribution.” We next conduct correlation analysis within each topic. This analysis aims to explore the relationship between sentiment and topics, providing a deeper insight into the sentiment distribution across different topics.

#### 4.4.1. Sentiment Distribution Calculation

After performing topic modeling and sentiment analysis, we calculated the sentiment distribution for each topic. Specifically, sentiment analysis was conducted with the RoBERTa model, categorizing text as either positive or negative, while BERTopic was used to assign topic labels to the text. By counting the number of positive and negative texts within each topic, we quantified the frequency of different sentiment categories within each topic, revealing the overall sentiment inclination of users towards specific topics.

To clarify the sentiment structure of each topic further, we calculated the proportion of positive and negative sentiments within each topic using the following formula:

(5) $$Sentiment\;Ratio=\frac{Number\;of\;positive\;(or\;negative)\;texts}{Total\;number\;of\;texts\;in\;the\;topic}\times 100\%$$

This ratio provides a more precise perspective on the sentiment distribution within each topic. By quantifying the sentiment distribution, we can identify sentiment characteristics for different topics, establishing a foundation for the subsequent correlation analysis. This analysis provides a clear understanding of the sentiment tendencies of each topic, enabling the quantification and comparison of user sentiment across different themes.

#### 4.4.2. Correlation Analysis of Topic Content and Sentiment Orientation

After analyzing the sentiment distribution across topics, similar topics were merged to improve the interpretability of topic modeling. The merging process was based on the semantic similarity of topics and keyword overlap. For instance, Topic 8, Topic 66, and Topic 101 all contain keywords related to the powertrain (e.g., “hybrid”, “battery”, “fuel consumption”, and “electric”), indicating discussions about automotive power systems, thus consolidated into a representative “Powertrain” topic. Similarly, Topic 31 and Topic 62 share keywords related to interior comfort (e.g., “seats”, “adjustment”, “front row”, and “back row”), so they were grouped under “Comfort and Interior.” Other topics were merged based on semantic content and keyword similarity, ensuring that the model’s output topics accurately reflect user discussions with enhanced interpretive power.

To validate the merging process and topic labeling, we conducted manual reviews of randomly sampled posts. This review was carried out by three experts with extensive experience in the automotive industry, ensuring that the assigned topic labels accurately reflected the underlying content. The experts evaluated the semantic consistency of each merged topic, confirming that the grouped posts shared a common theme and that the assigned labels were appropriate. Their involvement further validated the accuracy and reliability of the topic modeling results.

Subsequent to the aggregation, the sentiment distribution for each merged topic was recalculated by combining the sentiment proportions of the individual topics. The sentiment proportion for each merged topic was derived through a weighted average, accounting for the number of texts in each topic. This approach ensures that the sentiment distribution of the aggregated topics accurately reflects the sentiment trends of the original topics. The formula for calculating the weighted sentiment proportion is as follows:

(6) $$P_{positive,merged}=\frac{\sum_{i}^{n}\left(N_{i}\times P_{i}\right)}{\sum_{i}^{n}N_{i}}$$

where $P_{i}$ represents the positive sentiment proportion of the *i*-th original topic, *N_i_* denotes the number of texts in the *i*-th original topic, and *n* is the number of topics aggregated into the new category.

Certain topics exhibit a nearly equal split between positive and negative emotions, indicating a balanced divergence of user opinions. This is due to the presence of emotionally polarized words in the UGC data, with roughly equal quantities of both positive and negative terms. This approach ensures that the sentiment distribution of each merged topic, after recalculation, more accurately and comprehensively reflects user emotions within broader themes, maintaining consistency and enhancing the interpretability of emotional trends in automotive discussions.

Finally, the sentiment analysis and text count statistics for each topic, as shown in Table 6, revealed significant changes in the emotional distribution. To further validate the reliability of these findings, we compared the clustering results generated by BERTopic with those obtained using the k-means algorithm. Although both methods produced similar emotional distributions, BERTopic outperformed k-means in capturing contextual relationships through the pre-trained language model, resulting in clusters with higher semantic consistency.

Conversely, topics such as safety, design, driving experience, and after-sales exhibit a higher proportion of negative sentiment, with values of 80%, 75%, 62%, and 58%, respectively. Discussions around safety often involve significant dissatisfaction with aspects such as collision avoidance systems, airbag reliability, and emergency braking sensors, reflecting unmet user expectations for advanced safety technologies, which significantly influence consumer perceptions of safety.

Negative feedback in the design category focuses on styling and color choices, indicating that users have strong opinions about esthetic preferences, perceiving the current designs as lacking innovation or alignment with their tastes. Based on these results, automotive manufacturers can implement innovative design strategies and service enhancements for vehicle appearance and color. For example, by leveraging AI-driven trend analysis and crowdsourced design competitions, user feedback can be integrated into their iterative design process, fostering the development of more esthetically pleasing and personalized vehicle models. Additionally, manufacturers can use augmented reality (AR) technology to allow users to preview and customize vehicle exteriors in a virtual environment, further enhancing user engagement and satisfaction.

Similarly, the analysis of after-sales topics also reveals a high negative sentiment ratio, with issues such as long repair delays, highlighting the need for service quality improvements. In response, automotive manufacturers can enhance customer loyalty and satisfaction by extending warranty periods, offering free regular inspections, or providing other value-added services. Additionally, introducing intelligent scheduling systems and real-time tracking features would allow users to stay informed about the repair progress and reduce wait times.

For interior space and interior features, the positive sentiment ratios are 65% and 58%, respectively, indicating overall satisfaction with seat comfort, luxury design, and spaciousness. However, the 35% and 42% negative sentiment ratios also signal room for improvement, such as in enhancing the practicality and comfort of interior design to meet user expectations fully.

The sentiment ratios for noise control and price factors are both balanced at 50%, revealing a split in user opinion. For noise control, users appreciated advancements in noise insulation technologies, including noise sensors that mitigate wind and engine noise. However, others noted persistent issues with cabin noise during high-speed driving, highlighting the need for more robust acoustic control measures. Regarding pricing, feedback reveals significant polarization, with some users finding promotional offers and discounts appealing, while others criticize the perceived lack of value for money, suggesting the need for more transparent pricing strategies.

In driving experience, the negative sentiment ratio of 62% reflects dissatisfaction with handling, steering, and suspension systems. Additionally, feedback on lane-keeping sensors indicates concerns about their accuracy and reliability, particularly in urban and complex driving environments, where users expect the seamless integration of these features to enhance the overall driving experience.

Lastly, the eco-friendly category, with a high positive sentiment ratio of 86%, reflects strong user approval of the fuel economy and hybrid technologies. The inclusion of energy consumption sensors further demonstrates user interest in technologies that optimize energy use and improve environmental performance. However, the 14% negative sentiment reveals user concerns about the consistency of energy monitoring systems, suggesting opportunities for further refinement in this area.

## 5. Discussion and Limitations

### 5.1. Comprehensive Analysis and Discussions Regarding Automotive User Needs

**Figure 5:**
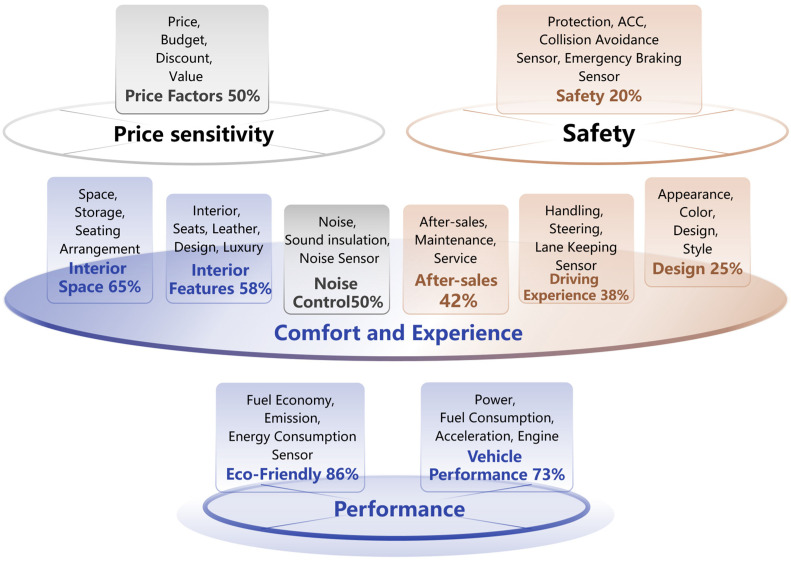
Diagram of user need categories and sentiment distribution.

Through the sentiment–theme association analysis of automotive users’ UGC data, we categorized user needs into four types: performance, comfort and experience, safety, and price sensitivity, as shown in Figure 5.

First, performance needs reflect users’ focus on fuel efficiency and vehicle performance, particularly regarding the powertrain, acceleration, and fuel consumption. Positive sentiment dominates in these topics (86% and 73%, respectively), especially in discussions about hybrid and electric vehicles. This indicates that most users appreciate advancements in power efficiency and expect further performance optimizations. Notably, the integration of energy consumption sensors further enhances users’ approval by providing more accurate real-time fuel efficiency monitoring, contributing to the growing preference for eco-friendly vehicles.

Second, comfort and experience needs cover a wide range, including the interior space, configuration, noise control, after-sales service, driving experience, and exterior design. Positive sentiment is relatively high for the interior space and configuration (65% and 58%, respectively), showing satisfaction with design, seat comfort, and layout. However, negative sentiment regarding materials and seating arrangements suggests a need for improvements to meet higher standards of comfort. Notably, noise control shows a balanced sentiment, while after-sales service, driving experience, and exterior design reveal predominantly negative sentiment (42%, 38%, and 25%, respectively), indicating general dissatisfaction.

Price sensitivity displays high emotional polarization, with an even distribution of positive and negative sentiments (50% each). Negative sentiments stem from dissatisfaction with current pricing, while positive sentiments are driven by satisfaction with promotions or cost-effective models. This distribution suggests that companies should consider flexible pricing strategies to better align with customer expectations.

In terms of safety needs, negative sentiment dominates (80%), reflecting users’ strong focus on collision protection and airbag configurations. Dissatisfaction stems from safety performance not meeting expectations, with a clear demand for higher safety standards and enhanced protection in real-world applications. Users also expressed concerns about the reliability of collision avoidance sensors and emergency braking systems, which are vital in ensuring safe driving experiences.

From the perspective of the sentiment distribution, each need type exhibits distinct positive and negative sentiment patterns. Performance and environmental concerns, which are associated with positive sentiment, highlight users’ approval of power and fuel efficiency. Especially with new energy vehicles, users show high recognition and expectations for eco-friendly performance, hinting at opportunities for innovation in energy-saving technologies. The addition of energy consumption sensors is positively acknowledged, as they provide users with more control over energy efficiency.

On the other hand, needs associated with high negative sentiment reveal user dissatisfaction in certain areas. For example, the exterior design topic has a high proportion of negative sentiment (75%), indicating a lack of satisfaction with current styling and color choices, with a preference for innovative designs and diverse color options. Negative sentiment in after-sales service points to unmet expectations regarding repair time and service quality, while safety concerns reveal a strong demand for reliable safety features and transparent crash test results. Additionally, dissatisfaction with lane-keeping sensors, particularly in complex driving environments, further contributes to the negative sentiment in driving experience.

In summary, the sentiment–theme association analysis based on UGC data clarifies user needs in terms of performance, comfort, price sensitivity, and safety. By quantifying sentiment tendencies, this analysis provides automotive companies with a solid basis for future product optimization and user experience improvement, ultimately aiding in market positioning and enhancing their competitive advantage.

### 5.2. Limitations and Future Work

Although this study presents significant findings, there are still areas for improvement. First, this study utilizes data from multiple sources, including Autohome, Dongchedi, Weibo, and Zhihu, and ensures the quality of multi-source data by standardizing fields and applying data cleaning and filtering techniques. However, due to the potential underrepresentation of certain demographic groups or geographic regions, the data collected from these platforms may not fully capture the diversity of user opinions or experiences. In future work, we plan to improve data quality by incorporating additional data sources, such as user reviews from other platforms or industry-specific databases, to further enhance the representativeness and robustness of the analysis.

Additionally, we employed a widely adopted binary sentiment analysis approach, effectively distinguishing sentiment tendencies across different topics while maintaining a good balance between accuracy and computational efficiency. Introducing multi-level sentiment classification could provide more granular insights and enhance the capture of emotional nuances. However, its implementation would require substantial adjustments in data annotation, model training, and sentiment intensity classification, potentially disrupting the overall experimental flow. In future work, we plan to explore sentiment intensity quantification methods, combining deep learning and sentiment lexicons to refine sentiment categorization across various topics.

Finally, this study presents a method for mining UGC data that can be applied to the automotive industry and other sectors, such as consumer electronics. For example, smartphone and wearable device manufacturers often analyze customer reviews to identify recurring issues related to durability, software updates, and ergonomic design. Researchers can extract valuable insights from UGC to guide hardware improvements, optimize software features, and refine product designs to better meet consumer expectations by employing similar topic modeling and sentiment analysis techniques. Due to the limitations of data availability and the specific focus of this study, we primarily concentrated on the automotive sector. Future research can extend this method to industries like consumer electronics to test its adaptability across different contexts.

## 6. Conclusions

This study proposes a novel user requirement mining method based on BERTopic and RoBERTa, supported by empirical analysis to thoroughly explore automotive user needs. By collecting UGC data from four major Chinese media platforms, applying BERTopic for topic extraction, RoBERTa for sentiment analysis, and performing an association analysis between sentiments and topics, this study categorizes user requirements into four main types: performance, comfort, price sensitivity, and safety. Additionally, ten key topic keywords were identified along with their corresponding sentiment tendencies. This approach efficiently combines deep-learning-based sentiment analysis techniques with topic modeling methods, offering an integrated framework for user need identification and quantitative analysis from text data. The integration of sensors, such as energy consumption and noise sensors, further enhances the relevance of specific user preferences, especially in performance and comfort areas, suggesting a potential direction for future user requirement mining research.

Moreover, results indicate distinct emotional responses across key attributes, namely performance, safety, comfort, and price, thereby offering a detailed overview of user needs. For instance, positive sentiment towards eco-friendly technologies like hybrid and electric vehicles underscores increasing support for energy-efficient solutions, while notable dissatisfaction with exterior design and after-sales service highlights areas ripe for innovation. These findings provide automotive manufacturers with valuable quantitative insights for performance optimization, energy efficiency improvements, and pricing strategies, and suggest that integrated sensor technologies can significantly enhance user satisfaction, particularly through advanced safety features such as collision avoidance and lane-keeping systems. Although our research offers promising hints for product and service enhancements, further validation by industry experts is required.

## Figures and Tables

**Table 1:** Data extraction fields for the automotive user demand mining platform.

Field Name	Field Annotation	Field Function
platformId	Platform ID	Used to distinguish the source of platform data
contentId	Article ID	Used to uniquely identify articles
title	Article Title	The main title of the article
content	Article Content	The thematic content of the article
likesCount	Number of Likes	The total count of likes or positive feedback
commentsCount	Number of Comments	Records user engagement and interest in article content

**Table 2:** Sample of regular keywords in automotive user demand mining.

Category	Regular Keywords
Brands	BMW|Mercedes-Benz|Audi|Tesla|Toyota|Honda|Ford|Volkswagen|Lexus|Volvo|Mazda|Porsche|Nissan|Chevrolet|Cadillac|BYD|Geely|Changan|Great Wall|Chery|NIO|Xpeng|Lixiang|Hongqi|Roewe|MG|GAC|Dongfeng|FAW|Haval|JAC|Wei|Baojun|Venucia|Bestune|Ora|SAIC|Bisu
Models	SUV|Sedan|Sports Car|Convertible|MPV|Pickup|Electric Vehicle|Plug-in Hybrid|Hybrid Power|New Energy Vehicle|Fuel Vehicle|Supercar|Hatchback|Sedan|Crossover|Mini Car
Related Terms	Engine|Gearbox|Brake|Suspension|Chassis|Tire|Interior|Seat|Steering Wheel|Center Console|Headlight|Battery|Electric Motor|Air Conditioner|Instrument Panel|Window|Headlight|Reversing Radar|Sunroof|Heated Seat|Onboard Charger|Fuel Consumption|Endurance|Control|Power|Comfort|Acceleration|Braking Distance|Noise|Emission|Cost-Effectiveness|Value Retention Rate|Body Stability|Driving Assistance|Automatic Driving|Four-wheel Drive System|Suspension Comfort|100 km/h Acceleration|Battery Life|Smooth Shifting|Noise Isolation

**Table 3:** Data sample display.

Title	Text Content	Platform Source	Likes Count	Comments Count
4 March 2024	What impact will the BYD Qin new car at 79,800 have on joint venture cars?	Zhihu	303	46
9 November 2023	I don’t know about others, but Tesla is worth a try.	Autohome	12	49
5 January 2024	The panoramic sunroof is really nice.	Dongchedi	127	345
11 March 2024	How is the Audi A5’s intelligent driving?	Weibo	68	122

**Table 4:** Model parameter settings.

Parameter	Value
batch_size	128
learning_rate	5 × 10^−5^
epoch	4
hidden_size	768

**Table 5:** Model performance comparison.

Model	Accuracy	Precision	Recall	F1-Score
RoBERTa	0.9091	0.8621	0.8929	0.8771
BERT	0.8734	0.8547	0.8612	0.8579

**Table 6:** Sentiment analysis of topics and text count statistics.

Topic ID	Keywords	Category	Positive Sentiment Ratio	Negative Sentiment Ratio	Text Count
1	Fuel Economy, Emission, Hybrid, Electric, and Energy Consumption Sensor	Eco-Friendly	86%	14%	3712
2	Power, Fuel Consumption, Acceleration, and Engine	Vehicle Performance	73%	27%	11,524
3	Space, Storage, and Seating Arrangement	Interior Space	65%	35%	3956
4	Interior, Seats, Leather, Design, and Luxury	Interior Features	58%	42%	8316
5	Noise, Sound insulation, Quiet effect, Wind noise, Engine noise, and Noise Sensor	Noise Control	50%	50%	4729
6	Price, Budget, Discount, and Value	Price Factors	50%	50%	6429
7	After-sales, Maintenance, and Service	After-sales	42%	58%	5382
8	Handling, Steering, Suspension, Feedback, and Lane Keeping Sensor	Driving Experience	38%	62%	5212
9	Appearance, Color, Design, and Style	Design	25%	75%	4263
10	Safety, Collision, Airbags, Protection, ACC, Collision Avoidance Sensor, and Emergency Braking Sensor	Safety	20%	80%	7673

## Data Availability

Data are available upon request from the authors. The data that support the findings of this study are available from the corresponding author upon reasonable request.
